# ^13^C labeling dynamics of intra- and extracellular metabolites in CHO suspension cells

**DOI:** 10.1186/1753-6561-7-S6-P43

**Published:** 2013-12-04

**Authors:** Judith Wahrheit, Averina Nicolae, Elmar Heinzle

**Affiliations:** 1 Biochemical Engineering Institute, Saarland University, D-66123 Saarbrücken, Germany

## Background

Isotope labeling techniques have become a most valuable tool in metabolomics and fluxomics [1]. In particular the dynamics of label incorporation provide rich information about metabolism. A thorough understanding of CHO metabolism is crucial for metabolic engineering and process optimization.

## Materials and methods

### Experimental set-up

CHO-K1 cells were cultivated in protein free TC-42 medium (TeutoCell, Bielefeld, Germany) in 250 ml baffled shake flasks. For the non-stationary experiment the cultures were inoculated at a start cell density of 2 × 10^6 ^cells/ml in a start volume of 120 ml. Four parallel cultivations were performed, two with 100% [U-^13^C_6_]glucose and two with 100% [U-^13^C_5_]glutamine, respectively. Extracellular samples were taken from all four cultivations every 6 h for cell counting and determination of extracellular metabolite concentrations and extracellular labeling dynamics. Intracellular samples were taken alternately from the two replicates. After 2 min, 10 min, 20 min, 30 min, 60 min, 2 h, 4 h, 6 h, 12 h, 18 h, 24 h, 30 h, 36 h, and 48 h, a sample of 5 ml cell suspension was quenched in 45 ml ice-cold 0.9% sodium chloride solution, centrifuged for 1 min at 2000 × g, washed once by rinsing the cell pellet with 50 ml ice-cold 0.9% sodium chloride solution, and frozen in liquid nitrogen. Intracellular metabolites were extracted in methanol and water by repeated freeze-thaw cycles, as described previously [2]. Extracts were dried in a centrifugal evaporator.

### Analytics

Cell counting and viability determination was carried out using an automated cell counter (Invitrogen, Darmstadt, Germany). Quantification of extracellular glucose, organic acids and amino acids via HPLC was carried out as described recently [3]. For determination of extracellular labeling dynamics, lyophilized supernatants were resolved in dimethylformamid (0.1% pyridine) and derivatized with MBDSTFA (Macherey-Nagel, Düren, Deutschland). Dried cell extracts were resolved in pyridine (20 mg/ml methoxylamine) and derivatized with MSTFA (Macherey-Nagel, Düren, Deutschland). Samples were analyzed by GC-MS. Unique fragments containing the whole carbon backbone were chosen for excreted extracellular metabolites and selected intracellular metabolites of the central metabolism.

## Results

We observed a monotonic cultivation profile during short-term cultivation for 48 h. Metabolic steady state was confirmed by exponential growth and constant metabolite yields. The two tracers, glucose and glutamine, were the major carbon sources. Lactate, alanine, glycine, and glutamate were excreted, all other metabolites were consumed. Although serine, aspartate, and glutamine were only consumed, we found significant extracellular labeling of these metabolites indicating simultaneous consumption and excretion.

Label incorporation into intracellular pyruvate and lactate was very fast on [U-^13^C_6_]glucose (mainly m3). Isotopic steady state in extracellular lactate was reached after 12 h. Labeling in pyruvate and lactate was also found using [U-^13^C_5_]glutamine as tracer (mainly m1) indicating a significant reflux from TCA cycle via anaplerotic reactions. Label incorporation into alanine was slower than for pyruvate and lactate and had a different labeling pattern. A significantly higher m2 fraction on labeled glucose indicates synthesis after pyruvate has entered the mitochondria. Significant labeling of serine and glycine was found on labeled glucose but not on labeled glutamine indicating the absence of gluconeogenesis.

Label incorporation into TCA cycle metabolites was fast on both tracers approaching steady state in citrate within 6 h of cultivation. Nearly identical labeling patterns were found for fumarate, malate and aspartate indicating a tight connection between these metabolite pools. After 24 h a metabolic shift takes place. Glutamine was synthesized in significant amounts. Labeling in TCA cycle metabolites decreased and labeling in pyruvate, lactate, and alanine further increased.

## Conclusions

We present the very first study of ^13^C labeling dynamics in CHO suspension cells. We were able to capture labeling dynamics in excreted extracellular metabolites as well as in intracellular organic acids and amino acids providing a representative overview of the central metabolism in CHO cells. Furthermore, we could draw some first qualitative conclusions. These transient labeling data is currently used in a non-stationary ^13^C metabolic flux analysis in order to obtain an in-depth understanding of CHO central metabolism, e.g. about reversibilities and the connection between glycolysis and TCA cycle.
